# Autologous Microfragmented Adipose Tissue Reduces the Catabolic and Fibrosis Response in an In Vitro Model of Tendon Cell Inflammation

**DOI:** 10.1155/2019/5620286

**Published:** 2019-12-05

**Authors:** Marco Viganò, Gaia Lugano, Carlotta Perucca Orfei, Alessandra Menon, Enrico Ragni, Alessandra Colombini, Paola De Luca, Pietro Randelli, Laura de Girolamo

**Affiliations:** ^1^Orthopedics Biotechnology Lab, IRCCS Istituto Ortopedico Galeazzi, Milan, Italy; ^2^Laboratory of Applied Biomechanics, Department of Biomedical Sciences for Health, Università degli Studi di Milano, Via Mangiagalli 31, 20133 Milan, Italy; ^3^1st Clinica Ortopedica, ASST Centro Specialistico Ortopedico Traumatologico Gaetano Pini-CTO, Piazza Cardinal Ferrari 1, 20122 Milan, Italy

## Abstract

**Background:**

Mesenchymal stem cells (MSCs) emerged as a promising therapy for tendon pathologies. Microfragmented adipose tissue (*μ*FAT) represents a convenient autologous product for the application of MSC-based therapies in the clinical setting. In the present study, the ability of *μ*FAT to counteract inflammatory processes induced by IL-1*β* on human tendon cells (TCs) was evaluated.

**Methods:**

Cell viability and proliferation were evaluated after 48 hours of transwell coculture of TCs and autologous *μ*FAT in the presence or absence of IL-1*β*. Gene expression of scleraxis, collagen type I and type III, metalloproteinases-1 and -3, and cyclooxygenase-2 was evaluated by real-time RT-PCR. The content of VEGF, IL-1Ra, TNF*α*, and IL-6 was evaluated by ELISA.

**Results:**

IL-1*β*-treated TCs showed augmented collagen type III, metalloproteases, and cyclooxygenase-2 expression. *μ*FAT was able to reduce the expression of collagen type III and metalloproteases-1 in a significant manner, and at the same time, it enhanced the production of VEGF, IL-1Ra, and IL-6.

**Conclusions:**

In this in vitro model of tendon cell inflammation, the paracrine action of *μ*FAT, exerted by anti-inflammatory molecules and growth factors, was able to inhibit the expression of fibrosis and catabolic markers. Then, these results suggest that the application of *μ*FAT may represent an effective conservative or adjuvant therapy for the treatment of tendon disorders.

## 1. Introduction

Tendon disorders represent a common condition in the field of musculoskeletal injuries. Current treatment options often lead to unsatisfactory results, with persistence of pain and reduced physical activity level [1, 2]. Among the conservative approaches, the use of mesenchymal stem cells (MSCs) from the bone marrow and adipose tissue emerged as a promising treatment, capable to counteract the pathological processes characterizing degenerative disease in the orthopedic field [3], gathering interest also in the treatment of tendon disorders. Indeed, successful applications of cultured MSCs were described for the treatment of tendon injuries in preclinical models [4]. The use of adipose-derived stem cells (ASCs), or autologous adipose-derived products, already showed efficacy in the context of tendon disorders in clinical and preclinical trials [5–8]. In vitro investigations demonstrated that the coculture of ASCs or *μ*FAT improved proliferation, viability, and collagen and VEGF production in tendon cells (TCs) [9, 10]. In addition, in an in vitro model of TC inflammation, cultured adipose-derived MSCs were able to reduce the production of proinflammatory mediators [11]. Nevertheless, translation of MSC-based therapies implies extensive manipulation for the production of cells in good manufacturing practice (GMP) conditions and it is limited by the high costs of production and the regulatory issues related to the advanced therapy medicinal products [12, 13]. As a consequence, different solutions for adipose- and bone marrow-derived MSC isolation at the point of care have been proposed in recent years, with encouraging results [5, 14]. In particular, microfragmented adipose tissue (*μ*FAT) represents a convenient compromise for the exploitation of MSCs in the clinical practice, avoiding extensive cell manipulation, and culture expansion [15]. *μ*FAT is easily obtained by commercially available devices from autologous lipoaspirate, and it has been demonstrated to exert a therapeutic activity in clinical and preclinical settings, providing positive functional outcomes in particular for the treatment of knee osteoarthritis [16–20]. The procedure of *μ*FAT production and injection is compatible with a single-stage surgery and may be combined with different surgical procedures. The aim of the present study was to investigate the paracrine effect of *μ*FAT on the proinflammatory and catabolic response of TCs to tnterleukin-1*β-* (IL-1*β*-) mediated inflammation. This molecule has been described as the primary mediator of the inflammatory status preventing tissue healing in rotator cuff injury [21], and previous reports showed that IL-1*β*-treated TCs upregulated the expression of catabolic enzymes and the production of inflammatory cytokines [22–24]. Thus, the working hypothesis of the present study is that autologous *μ*FAT would counteract the catabolic and proinflammatory response elicited by IL-1*β* on TCs, providing insights on the mechanism of action of this therapeutic approach and strengthening the rationale of its use in tendon disorders.

## 2. Materials and Methods

### 2.1. TCs and *μ*FAT Isolation and Harvesting

Long head of biceps tendon biopsies and *μ*FAT were collected from 8 patients (5 females and 3 males, mean age: 60 ± 10 y/o) undergoing arthroscopic rotator cuff repair augmented with *μ*FAT. An informed consent was obtained from all patients as per the protocol approved by the Institutional Review Board (no. 148/INT/2015, January 13, 2016). Lipoaspirate adipose tissue was collected before surgery and processed by Lipogems® device, following the manufacturer's instruction [25]. An aliquot of *μ*FAT was harvested for in vitro experiments. Briefly, each *μ*FAT sample was centrifuged at 376 × *g* for 5 minutes at room temperature to separate the aqueous phase and the fragmented tissue. The former was stored at -20°C, while the latter was frozen at -80°C after the addition of 1 volume of freezing solution (90% FBS, 10% dimethyl sulfoxide; Sigma-Aldrich, St. Louis, MO, USA). Separation of the two phases was adopted to maintain the composition of injected *μ*FAT even after thawing and removal of the aqueous freezing solution. This allowed for the use in the experiment of an identical product as injected during the surgical procedure.

Human tendon cells (TCs) were isolated from long head of the biceps tendon biopsies obtained during arthroscopic rotator cuff repair. Tendon tissue was digested with a 0.3% *w*/*v* collagenase type I solution (Worthington, Lakewood, NJ, USA) for 16 hours at 37°C, as previously reported [26]. TCs were cultured in a complete medium composed by high-glucose Dulbecco's modified Eagle's medium (DMEM; Sigma-Aldrich) with 10% *v*/*v* fetal bovine serum (FBS; Euroclone, Pero, Italy), 100 U/ml penicillin, 100 *μ*g/ml streptomycin, 0.29 mg/ml L-glutamine (Gibco, Waltham, MA, USA), and 5 ng/ml bFGF (Peprotech, London, UK). The medium was replaced every 3 days, and all the experiments have been performed at passage 3. After culture, TCs represent a mixed population of terminally differentiated and tendon progenitor cells at different stages of differentiation, as previously reported [26, 27].

### 2.2. Treatment of TCs with IL-1*β* and Coculture with *μ*FAT

TCs were seeded in 12-well plates at the density of 50000 cells/well. TCs were either maintained in normal culture medium or a medium added of 1 ng/ml IL-1*β*. In addition, cells treated with IL-1*β* and untreated cells were cocultured with 250 *μ*l of *μ*FAT, made of125 *μ*l of microfragmented tissue and 125 *μ*l of the corresponding aqueous phase. The two phases were separated in order to maintain the aqueous part of *μ*FAT, even after thawing and removal of the freezing solution. *μ*FAT was added to the top portion of a transwell insert (pore size: 0.4 *μ*m), while TCs were seeded on the bottom. Equal volume of phosphate-buffered saline (PBS) was added in the transwell top portion of the samples without *μ*FAT. After 48 hours of treatment, the culture media were collected and stored at -20°C, while cells were trypsinized and the pellets stored at -80°C.

### 2.3. RNA Isolation and Gene Expression

RNA was obtained from cell pellets using TRI reagent (Sigma-Aldrich). Briefly, cells were lysed by the addition of 300 *μ*l TRI reagent, and then 100 *μ*l of 1-bromo-3-chloropropane (Sigma-Aldrich) were added to the samples. After centrifugation at 12000 × *g* for 10 minutes, the interphase was collected for DNA extraction while the RNA in the aqueous phase was precipitated with isopropanol, washed in 75% ethanol and then resuspended in 20 *μ*l RNAse-free water for storage at -80°C.

Total RNA from each sample was transcribed to cDNA using iScript™ cDNA Synthesis Kit (Bio-Rad Laboratories, Hercules, CA, USA) as per manufacturer's instructions. Real-time PCR was performed starting from 10 ng of cDNA, using a PCR mix containing TaqMan® Universal PCR Master Mix and Assays-on-Demand Gene expression probes (Life Technologies, Waltham, MA, USA) for *SCX* (scleraxis; Hs03054634_g1), *COL1A1* (collagen type I alpha 1 chain; Hs01076777_m1), *COL3A1* (collagen type III alpha 1 chain; Hs00943809_m1), *MMP1* (metalloproteinase-1; Hs00899658_m1), *MMP3* (metalloproteinase-3; Hs00968305_m1), and *PTGS2* (cyclooxygenase-2; Hs00153133_m1). Applied Biosystems StepOnePlus® (Life Technologies) was used to perform all experiments (program: 1 cycle of 2 minutes at 50°C, 1 cycle of 10 minutes at 95°C, 40 cycles of 15 seconds at 95°C, and 1 minute at 60°C). The results were normalized against the mean expression of two housekeeping genes: *YWHAZ* (tyrosine 3-monooxygenase/tryptophan 5-monooxygenase activation protein zeta, Hs03044281_g1) and *ACTB* (*β*-actin, Hs99999903_m1) identified in a previous study [28]. Two replicates were analyzed for each sample, and data were presented according to the ΔΔ**Ct** method [29].

### 2.4. DNA Isolation and Quantification

DNA was separated from the interphase (obtained as described above) by the addition of 100% ethanol and centrifugation at 2000 × *g* for 5 minutes. The DNA pellet was washed in 0.1 M trisodium citrate, 10% ethanol solution for 30 minutes, and then centrifuged at 2000 × *g* for 5 minutes. DNA was then washed in 75% ethanol, and solubilized in 50 *μ*l 8 mM NaOH for storage at -20°C. DNA content in each sample was measured by NanoDrop spectrophotometer at 260 nm absorbance using software version 3.7.1 (NanoDrop ND-1000, ThermoFisher Scientific) [30].

### 2.5. TC Metabolic Activity

Viability was assessed by alamarBlue assay after 48 hours of treatment (ThermoFisher Scientific, Waltham, MA, USA). Briefly, culture medium was removed and cells were incubated with a 10% *v*/*v* solution of alamarBlue in DMEM at 37°C. After 2 hours of incubation, fluorescence was measured by a spectrophotometer (Victor X3, Perkin Elmer, Waltham, MA, USA) with excitation of 540 nm and emission of 590 nm.

### 2.6. ELISA

The release of interleukine-6 (IL-6), interleukine-1 receptor antigen (IL-1Ra), tumor necrosis factor *α* (TNF***α***) (Peprotech), and vascular endothelial growth factor (VEGF) (R&D Systems, Minneapolis, MN, USA) in the culture media of TCs treated for 48 hours was analyzed by ELISA assays, following the manufacturer's instruction. The detection ranges were as follows: 24–1500 pg/mL for IL-6, 23–1500 pg/mL for IL-1Ra, 31-2000 pg/ml for TNF*α*, and 31.3-2000 pg/ml for VEGF.

### 2.7. *μ*FAT Cell Count and Viability Assays

After thawing, an aliquot of *μ*FAT was digested by 0.075% *w*/*v* collagenase type I (Worthington) for 45 minutes at 37°C. The number of cells and viability was assessed by NucleoCounter NC-3000 using cell viability staining (Chemometech, Allerod, Denmark) [31]. The *μ*FAT samples used in the study had a mean cell count of 2.3 ± 1.3 × 10^6^ cells/ml, while cell viability was 53.2 ± 13.1%.

### 2.8. Statistical Analysis

**Figure 1:**
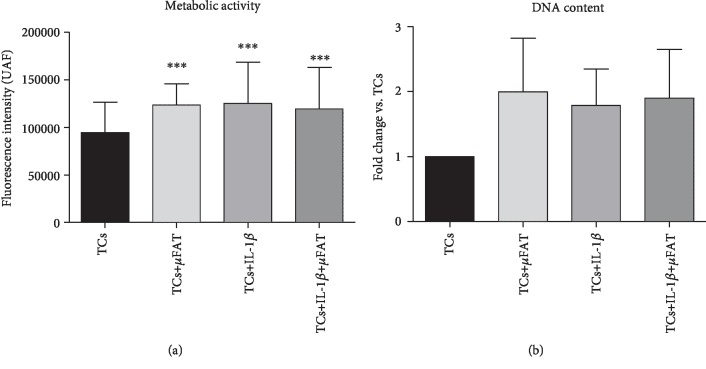
Metabolic activity (a) and DNA content (b) of untreated and differently treated TC samples (*n* = 8). ^∗^*p* < 0.05, ^∗∗∗^*p* < 0.001 vs. TCs.

**Figure 2:**
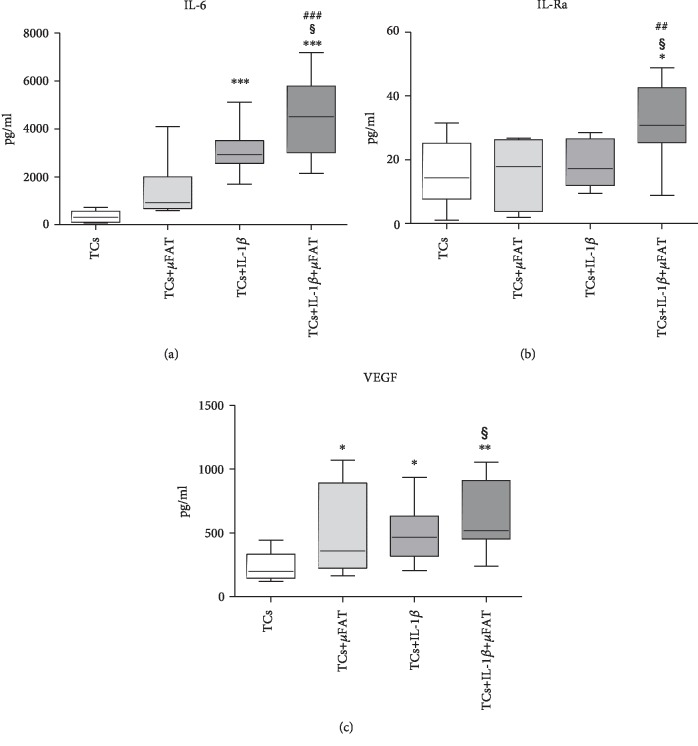
IL-1Ra, IL-6, and VEGF quantification in culture media of TCs treated with IL-1*β* and/or *μ*FAT and untreated TCs (*n* = 8). ^∗^*p* < 0.05, ^∗∗^*p* < 0.01, ^∗∗∗^*p* < 0.001 vs. TCs; ^##^*p* < 0.01, ^###^*p* < 0.001 vs. +*μ*FAT; ^§^*p* < 0.05 vs. +IL-1*β*.

**Figure 3:**
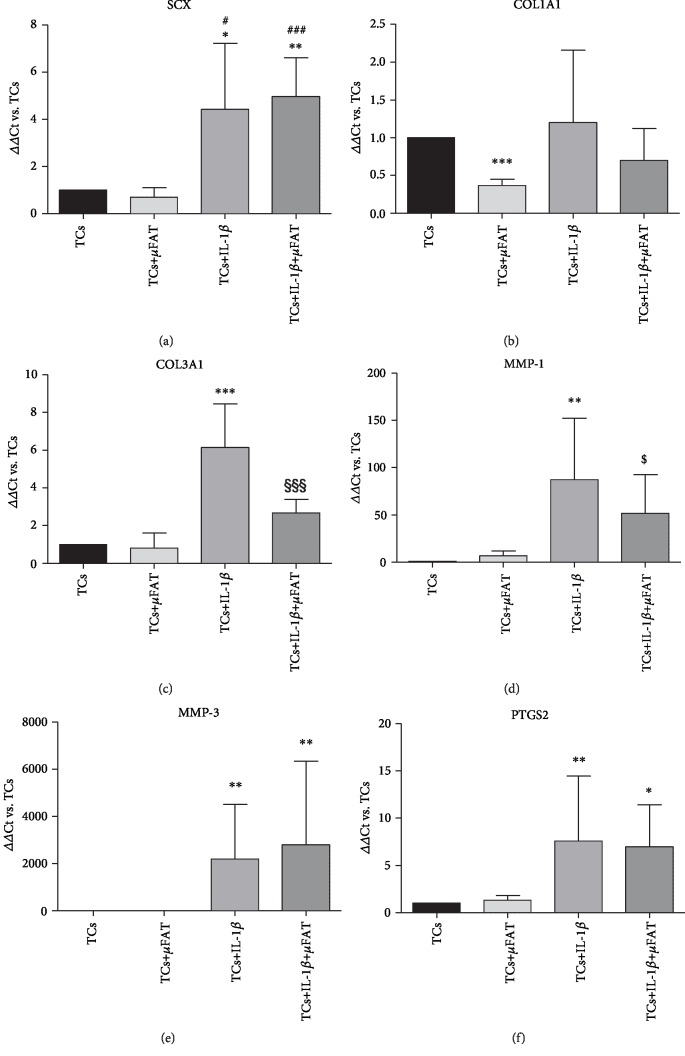
Gene expression of *SCX*, *COL1A1*, *COL3A1*, *MMP1*, *MMP3*, and *PTGS2* in TCs cultured in the presence of IL-1*β* and/or *μ*FAT (*n* = 8). Data are expressed as mean ΔΔCt with respect to untreated controls (TCs = 1). ^∗^*p* < 0.05, ^∗∗^*p* < 0.01, ^∗∗∗^*p* < 0.001 vs. TCs; ^#^*p* < 0.05, ^###^*p* < 0.001 vs. +*μ*FAT; ^§§§^*p* < 0.001 vs. +IL-1*β*; ^$^*p* < 0.1 vs. +IL-1*β* (tendency).

All the analyses were performed using Prism 5.0 (Graphpad Software, La Jolla, CA, USA). Gaussian distribution of data was assessed by Shapiro-Wilk test. One-way repeated measures ANOVA test with Bonferroni's post test was applied to measure the differences among treatments when data presented a Gaussian distribution (Figures 1(a), 2(b), and 2(c)); otherwise, the Friedman's test with Dunn's post-test was used (Figures 1(b), 2(a), and 3). A level of *p* < 0.05 was considered statistically significant.

## 3. Results

### 3.1. IL-1*β* and *μ*FAT Enhance TC Metabolic Activity

The metabolic activity of IL-1*β*-treated TCs was significantly increased with respect to untreated TCs (*p* < 0.01), and no further improvement was induced by the coculture with *μ*FAT. In noninflammatory conditions, samples cocultured in transwell with *μ*FAT demonstrated a higher metabolic activity with respect to untreated TCs (*p* < 0.05) (Figure 1(a)). DNA content resulted in increased in all conditions, even if in a nonsignificant manner (Figure 1(b)).

### 3.2. *μ*FAT Reduces the Gene Expression of Catabolic and Fibrosis Markers

The presence of IL-1*β* significantly enhanced the TC expression of *SCX* (*p* < 0.05), *MMP1* (*p* < 0.01), *MMP3* (*p* < 0.01), *COL3A1* (*p* < 0.001), and *PTGS2* (*p* < 0.01) with respect to untreated TCs, while it did not exert any effect on *COL1A1* expression (Figure 3). In this inflammatory condition, the coculture with *μ*FAT decreased the expression of *COL3A1* (-57%, *p* < 0.001) and *MMP1* (-41%, *p* = 0.08) (Figures 3(c) and 3(d)), with little effect on the other parameters. No effect of *μ*FAT on *MMP3* expression was observed, while it was strongly induced by IL-1*β* treatment (Figure 3(e)). Moreover, TCs cocultured with *μ*FAT, with or without IL-1*β*, showed a reduced expression of *COL1A1* with respect to untreated TCs (*p* = 0.06 and *p* < 0.001 in the presence or absence of IL-1*β*, respectively) (Figure 3(b)). A slight increase in *SCX* expression was observed in TCs cocultured with *μ*FAT and treated with IL-1*β*, with respect to IL-1*β* only treated cells (+12%, n.s.) (Figure 3(a)).

A fivefold increase in overall *COL3A1*/COL1A1 expression ratio was observed in IL-1*β*-treated TCs with respect to untreated TCs (*p* < 0.05, data not shown). In inflammatory conditions (+IL-1*β*), the coculture with *μ*FAT was able to reduce the ratio, even if in a nonstatistically significant manner (-27%, n.s.), while in TCs+*μ*FAT, the ratio was increased in a nonsignificant manner in comparison to untreated TCs samples, in both inflammatory and noninflammatory conditions.

### 3.3. Il-1*β* and *μ*FAT Enhance the Production of Cytokines and VEGF

In the presence of IL-1*β*, an enhanced production of IL-6 (*p* < 0.001) was observed with respect to untreated TCs, and it was further increased by the coculture with *μ*FAT (+45%, *p* < 0.05 with respect to TCs+IL-1*β*) (Figure 2(a)). On the contrary, IL-1Ra production was not induced by IL-1*β* or *μ*FAT alone, while the contemporary presence of these factors significantly increased the production of this molecule (+217% and +290%, with respect to both untreated TCs and IL-1*β*-treated TCs; *p* < 0.05) (Figure 2(b)). The production of VEGF was induced by IL-1*β* (+109% with respect to untreated cells, *p* < 0.05) or *μ*FAT coculture (+116% with respect to untreated cells, *p* < 0.05); the combination of both factors further increased VEGF production (+29% in comparison with TCs treated with IL-1*β*, *p* < 0.05) (Figure 2(c)). The content of TNF*α* was undetectable in all samples (data not shown).

## 4. Discussion

The present study aimed to test the ability of autologous *μ*FAT in favouring tissue healing in the context of tendon cell inflammation. Our results demonstrated that the paracrine action of *μ*FAT effectively reduces the expression of catabolic and fibrosis markers in pair-matched TCs cultured in inflammatory conditions. The strength of this work is the use of patient-matched TCs and *μ*FAT, allowing for taking into account the inter-donor variability in both elements.

More in details, in cells exposed to IL-1*β*, the paracrine action of *μ*FAT was able to significantly reduce the *COL3A1/COL1A1* ratio which positively correlates with the deposition of rupture-prone fibrotic tendon matrix [32, 33], confirming previous findings obtained in cocultures of TCs and adipose-derived MSCs [34, 35]. IL-1*β* has been described as the key effector of tendon inflammation leading to the inhibition of the proper healing process [21, 23, 24], also enhancing the production of catabolic enzymes such as MMP1 and MMP3 involved in tendon matrix degradation [36, 37], and increasing collagen type III expression over collagen type I. Moreover, IL-1*β* directly induces the production of the enzyme cyclooxygenase-2 (COX-2), typical marker of inflammation [38]. Interestingly, cells cultured in inflammatory condition in presence of *μ*FAT showed an overall reduction of the absolute expression of both collagen type I and III. The increase in collagen type III after injury is associated with the formation of scar tissue, which allows for prompt healing at the expense of tissue quality and functionality [39, 40]. In this view, a slower matrix deposition, where the proportion between the different types of collagen is similar to the physiological level, represents a therapeutic goal aimed to improve the quality of regenerated tissue.

In addition, *μ*FAT was able to inhibit *MMP1* expression, suggesting a protective role towards tendon ECM integrity. Metalloproteases are crucial for the physiological maintenance of tendon extracellular matrix (ECM) homeostasis [41], to the extent that the inhibition of MMPs results in pathological changes and pain [42]. On the other hand, an excess of MMP expression is associated with aberrant matrix degradation, typical of degenerative tendon disorders [43]. In particular, MMP1 is clearly involved in tendon pathology in association with the action of IL-1*β* [44, 45]. The effect of *μ*FAT on MMP expression is consistent with previous observations reported in a model of inflamed synoviocytes, supporting the inhibition of MMP-related matrix degradation as a mechanism of action of this product [46]. Moreover, when inflammatory processes are not involved, *μ*FAT has little effect on the expression of MMPs, indicating that it would not inhibit the physiological remodeling of tendon ECM.

In this study, the influence of IL-1*β* on the expression of the tendon-specific transcription factor *SCX* was also assessed. While previous reports showed an inhibition of its transcription in inflammatory conditions [23, 47, 48], in our model, the presence of IL-1*β* significantly increased *SCX* expression. The discrepancy between the results described in literature and those reported in the present study is probably due to the different sources of TCs and to the inflammatory protocol. Since adipose-derived MSCs are known to produce trophic mediator-specific for TCs [49], a slight reduction of *SCX* expression was observed when TCs were cocultured with *μ*FAT in basal conditions, and a downregulation of this marker have been already reported by other authors after TCs-MSCs coculture [34]. These observations suggest that *μ*FAT (or MSCs) trophic action on TCs may be independent of *SCX* upregulation.

Our findings showed no effect of *μ*FAT on *PTGS2* expression, neither in inflammatory nor in noninflammatory conditions. As expected, *PTGS2* transcription was enhanced by IL-1*β* treatment with respect to basal conditions, being IL-1*β* the main inducer of this gene encoding for COX2 [38]. Despite its role in inflammation, COX2 has been reported exerting an important role in the maintenance of tendon homeostasis and ECM maturation [50, 51]. Therefore, the expression of this enzyme may represent not only as a symptom of inflammation but also as a response of TCs towards tissue healing.

IL-1*β* and *μ*FAT both demonstrated an enhancing effect on TC metabolic activity and DNA content, even if no statistical significance was found for the latter. Interestingly, this effect was not further enhanced by the use of IL-1*β* on TCs cocultured with *μ*FAT, showing a lack of additive/synergistic action of the two elements on these parameters.

In our model, due to the use of a transwell system, the modifications induced by *μ*FAT on TC gene expression and metabolic activity are ascribed to its paracrine activity. In particular, *μ*FAT increased the content of soluble IL-1Ra, IL-6, and VEGF in the culture medium. IL-1Ra is a direct inhibitor of IL-1*β*, and it acts by competing with the cytokines for the binding to IL-1 receptor 1 [52], to the extent that IL-1Ra-based treatments have been developed for rheumatoid arthritis and other autoinflammatory diseases [53]. IL-1Ra production was elicited in *μ*FAT samples only in the presence of inflammatory conditions, suggesting it is a reaction of the *μ*FAT-embedded adipose-derived MSCs to this condition. Indeed, these cells are known to respond to IL-1*β* stimulation by releasing IL-1Ra [54], as well as MSCs from other sources [55].

As expected, in our model, the content of IL-6 was enhanced in the presence of IL-1*β* [56] and *μ*FAT further increased its production. This effect is possibly related to the IL-1*β*-mediated induction of IL-6 in the cells contained in *μ*FAT [54], and even if there are little evidences of direct pathological changes mediated by IL-6 on TCs and tendon matrix production [57], this aspect should be taken into account as a possible side effect when applying cell-based regenerative medicine products. The role of IL-6 in tendon pathologies has been confirmed by several studies, and it is considered one of the evidences of inflammation involvement in tendinopathy [58]. Indeed, IL-6 increased after tears and ruptures, as well as after intense exercise and injuries, in both humans and animals [59]. Nevertheless, despite its role in inflammation, IL-6 exhibits an immunoregulatory activity and it demonstrated to support tenocyte proliferation and survival, thus resulting among the effectors of the early phases of tendon healing [60–62]. In addition, IL-6 induces the production of VEGF [63], a growth factor mainly known for its role in angiogenesis, that is also involved in tissue regeneration, exerting a homeostatic function [64]. Different to what is observed in IL-6, VEGF is induced by both IL-1*β* and *μ*FAT treatments at similar intensities, and it was further enhanced by the combination of these factors. Then, since IL-6 was just slightly enhanced by *μ*FAT treatment in comparison to untreated TCs, the *μ*FAT-induced VEGF production appears to be at least partially independent from the IL-6 pathway. Despite the promotion of angiogenesis which may result detrimental for tendon ECM integrity [65], VEGF was able to improve tendon healing strength in several studies [66–68].

The ability of MSCs and *μ*FAT paracrine mediators to counteract inflammation is well described, and it has been reported in several models involving different cells and tissues, comprising chondrocytes, synoviocytes, and the central nervous system [46, 69, 70]. Indeed, the increased production of VEGF and the augmented expression of *SCX* and, to some extent, *COL3A1*, demonstrate that IL-1*β* also elicits an initial reparative response in TCs [71]. This observation is consistent with the presence of a subpopulation of progenitor cells, demonstrating features of mesenchymal stem/stromal cells, within TCs [26, 72]. In pathological conditions and aging, the presence of progenitor cells may be reduced, leading to a loss of homeostatic function and thus tissue degeneration [73]. Then, the idea to supply the injured tissue with a convenient source of autologous MSCs with homeostatic and immunomodulatory activity perfectly represents the rationale of the application of cell concentrates in degenerative disorders.

Limitations of the present study are represented by the use of a nonphysiological inflammatory stimulus that may result in considerably stronger than the one occurring in vivo, and thus partially masking the ability of *μ*FAT to counteract the catabolic response. In addition, the quantity of *μ*FAT was chosen based on previous reports [9], but the proportion between cells and tissue hardly corresponds to the physiological ratio. Another limitation is given by the possible influence of patients' characteristics on the activity of *μ*FAT, which was not taken into account in the present work while it may indeed identify a fraction of individuals who are nonresponders to the *μ*FAT treatment due to pathology or adipose tissue-specific features [74] and thus representing a source of bias.

The results obtained in this work sustain the rationale of *μ*FAT application to tendon disorders and they may provide evidences for the interpretation of future clinical data, identifying the possible mechanisms involved in *μ*FAT-mediated effects on tendon healing. A clinical trial is now ongoing for the evaluation of the possible clinical benefit of *μ*FAT injection as adjuvant therapy in the arthroscopic rotator cuff repair, especially in terms of possible reduction of retears.

## 5. Conclusions

In an inflammatory context, TCs demonstrated a catabolic and profibrosis pattern of gene expression, while at the same time, producing molecules with a role in tissue healing. The coculture with *μ*FAT not only reduced the expression of fibrosis and catabolic markers but it also enhanced the production of cytokines and growth factors able to counteract the inflammatory process and to contribute to tissue regeneration. These observations provide a rationale for the clinical application of *μ*FAT in the treatment of tendon disorders.

## Data Availability

All data used to support the findings of this study are available from the corresponding author upon request.
